# Blood Cadmium Level Is Associated with Short Progression-Free Survival in Nasopharyngeal Carcinoma

**DOI:** 10.3390/ijerph16162952

**Published:** 2019-08-16

**Authors:** Taifeng Du, Wenlong Huang, Shukai Zheng, Mian Bao, Yuanni Huang, Anna Li, Meirong He, Kusheng Wu

**Affiliations:** Department of Preventive Medicine, Shantou University Medical College, Shantou 515041, China

**Keywords:** Nasopharyngeal carcinoma, progression-free survival time, blood cadmium levels, clinical characteristics, survival analysis

## Abstract

The prognosis of nasopharyngeal carcinoma (NPC) is poor with disease progression. Cadmium exposure is a risk factor for NPC. We aimed to investigate the effect of cadmium exposure, by measuring cadmium level, and clinicopathologic factors on NPC disease progression and prognosis. A total of 134 NPC cases were analyzed and venous blood samples were collected. Blood cadmium level was analyzed by graphite furnace atomic absorption spectrophotometry. Clinical data were collected at baseline for patients and tumor characteristics from medical records. Progression-free survival (PFS) was analyzed during follow-up. The effect of cadmium exposure and clinical factors on PFS was analyzed by the Kaplan–Meier method and Cox regression models. Blood cadmium level was associated with history of disease and smoking history and pack-years. On Kaplan–Meier analysis, a high blood cadmium level, male sex, smoking history and increasing pack-years, as well as advanced clinical stage were all associated with short PFS. On multivariate analysis, blood cadmium level was an independent risk factor and predictor of NPC prognosis and disease progression. Cadmium exposure and related clinical factors can affect the prognosis of NPC, which merits further study to clarify.

## 1. Introduction

Nasopharyngeal carcinoma (NPC) occurs in epithelial cells of the nasopharynx and belongs to head and neck cancers. Although NPC has similar tissue cells and blood systems as epithelial neoplasms, the two cancers differ in the head and neck region. The incidence of NPC is approximately 87,000 patients/year worldwide, and approximately 50,000 die from NPC annually [1]. As compared with other tumors, NPC is not common and features a geographical distribution trend. The first study of 14 patients combined NPC patients and was published in 1901 [2]. Then, a comprehensive study of 114 patients with a combination of clinical features was published in 1941 [3]. In the United States and Europe, the incidence of NPC is 0.5 to 2/100,000 people [4]. A higher incidence, approximately 80/100,000 people, has been reported in South Asia, parts of the Mediterranean and North Africa [5]. NPC is endemic in Guangdong province, southern China, with an incidence of up to 20 to 30/100,000 people [6]. 

The etiology of NPC is multifactorial and may be interactive. Possible risk factors include eating salted fish, smoking tobacco, drinking alcohol, exposure to fumes, etc. [7,8,9]. However, these daily behavioral factors and lifestyles are not sufficient to explain the high incidence of NPC, so there are other unknown risk factors. Occupational exposure to asbestos and cement dust, for example, may increase the risk of NPC [10,11]. Metals are involved in the metabolism of the body for maintaining normal life activities. However, an imbalance in element contents can damage the human body. Long-term occupational exposure to heavy metals and smoking can increase heavy metal levels in the blood [12,13]. Tobacco contains many harmful substances, including heavy metals [14,15], which are poisonous and carcinogenic. 

Many previous studies found that environmental exposure and occupational exposure to metals are associated with a variety of human cancers. Specifically, decreased selenium (Se) and zinc (Zn) levels may be related to cervical intraepithelial neoplasia and invasive cancer [16]; cadmium (Cd) levels are positively correlated with risk of lung cancer in males [17] and mouth cancer in females [18]; risk of oral cancer is increased with nickel and chromium levels [19]; inorganic arsenic in drinking water is associated with lung and bladder cancers in males [20]; and trace elements have a significant role in the development of carcinoma of larynx [21].

Cd, as a toxic metal, has not been specifically studied and may be involved in the development of NPC [22]. Cd mainly originates from industrial processes such as mining and metal smelting and textile production [23]. People are exposed to Cd mainly through natural contact (such as food, water and dust-containing metal ions) and occupational contact [24]. For non-occupationally exposed people, the primary source of Cd is diet and smoking tobacco [25]. Tobacco is also an important source of chronic Cd exposure. The human body absorbs approximately 3 μg Cd by lung absorption when smoking a pack of cigarettes per day [26]. Long-term exposure to Cd causes harm to human health when the accumulation of elements in human body increases beyond the degradation ability of the body and it is difficult to excrete them. Previous studies have found that higher Cd levels are associated with the occurrence of other types of cancer, such as endometrial cancer [27], bladder cancer [28], pancreatic cancer [29], and postmenopausal breast cancer [30]. Low to moderate Cd exposure was prospectively associated with total cancer mortality of the lung and pancreas [31]. Therefore, the harm of Cd to the human body and the occurrence of tumors is a concern.

Although research on the pathogenesis of Cd in the body is relatively scarce, because of its long-term accumulation, Cd level is likely highly correlated with the prognosis of a tumor such as NPC and its progression. Many factors affect disease progression of NPC, and there may be an interaction between various factors. Disease progression refers to a 20% increase in the sum of the longest diameters of all target lesions and is not considered with increase in size of just one lesion. In addition to the expansion of local lesions, the other type of disease progression is transfer to another part. These two situations can be found mainly on imaging. Actually, clinical deterioration belongs to disease progression and is characterized by tumor spread and metastasis, disease recurrence, nosebleed, and lymph node transfer in the head and neck. The potential factors affecting the survival and prognosis of NPC can generally be divided into internal and external factors. The status of tumor cells themselves such as the nature of tumor cells and surrounding stroma may also be an important factor for clinical progression [32]. Moreover, previous studies showed that clinical stage, sex, smoking history and T classification are significantly associated with the survival and prognosis of NPC [33,34,35]. These factors may be related to distant metastasis and progression of NPC.

Previous studies have found that Cd level is possibly associated with the development of NPC [22,36,37]. In the present study, to explore the prognostic factors of NPC and further predict NPC progression, clinical information and baseline data were collected and the blood Cd level of patients was measured to explore the possible associations between these factors and NPC progression and prognosis.

## 2. Materials and Methods

### 2.1. Subjects

In December 2014, from our medical records, we selected patients with a diagnosis of NPC from 1 August 2002 to 18 February 2014 at the Cancer Hospital in Shantou city. We included patients who were (1) receiving similar treatment (cisplatin and paclitaxel chemotherapy), (2) had no occupational exposure to Cd, and (3) had histopathologically confirmed NPC. The incidence of NPC is as high as 30/100,000 people in southern China, especially in Guangdong province [6], so most of the patients included were from Chaoshan area, which is located in the eastern coastal area of Guangdong province, and the rest were from neighboring areas. After being informed of the specific purpose of the study, all patients were asked to complete an informed consent form. Blood samples were taken within 3 days after the histopathologic diagnosis of NPC. When informed consent was obtained, 2 mL venous blood was obtained from patients by a trained nurse, then put into K-EDTA (ethylene diamine tetraacetic acid) metal-free vials, and stored at −80 °C. All plastic tubes and containers used for the determination of blood Cd levels were washed, soaked in nitric acid, washed for deionization and dried before use to avoid contamination. The study was approved by the human ethics committee of Shantou University Medical College. The medical records and pathology reports of patients are available if needed.

### 2.2. Collection of General and Clinical Data

Relevant general, pathological and clinical data were obtained from medical records following the procedures and guidelines of the Cancer Hospital in Shantou city. General information included age, sex, smoking, alcohol drinking, residence, occupation, etc. Relevant general information was obtained from medical records and by face-to-face and telephone interview. For the cumulative smoking dose, we divided patients into four groups by pack-years of smoking: no smoking, <30, 30 to 40, and ≥40 pack-years. Clinical data included age of diagnosis, family history of cancer, and history of disease (including hypertension, diabetes, asthma, kidney stones, etc.). Pathological information mainly included tumor–node–metastasis (T, N, M) classification; Epstein–Barr virus antibody levels (early antigen (EA), viral capsid antigen antibody (VCA)); clinical stage; and pathological type.

T, N and M classification were classified according to the TNM system of the International Union for Cancer Control and the American Joint Committee on Cancer. T classification is divided into four groups by site of tumor spread: T1 (limited to the nasopharynx), T2 (spread to the nasal tissue), T3 (spread to bony structures), and T4 (spread to the brain or brain nerve). N classification is divided into four groups by the diameter of lymph nodes metastasized: N0 (no lymph node metastasis), N1 (largest diameter ≤3 cm), N2 (3–6 cm), and N3 (>6 cm). M classification is divided into two groups by whether the tumor has distant metastasis: M0 (no distant metastasis) and M1 (distant metastasis).

### 2.3. Collection of Survival Time Data

All patients were interviewed after diagnosis and treatment at the cancer hospital in Shantou city. We collected patient information from medical records. To conduct a follow-up, we set the baseline time of NPC diagnosis from 1 August 2002 to 18 February 2014 and the endpoint as 31 December 2014. Disease progression was defined as the disease recurrence, metastasis or death. Survival time was defined as the cancer-free period from the start of diagnosis and treatment at the hospital to the onset of disease progression. If no disease progression was observed at the endpoint, these data were censored. During the follow-up period, the survival time was defined as full data if the patient died or disease progression occurred. Survival time was a significant indicator for evaluating disease progression.

### 2.4. Analysis of Blood Cd Level

Blood samples were used to analyze the risk of Cd exposure in patients. Blood Cd levels were measured by a standardized method also used in other publications [38]. A volume of 200 μL blood sample was placed in a tube containing 800 μL 5% nitric acid. After vortexing, the sample was digested for 10 min, then centrifuged for 15 min at 3000 rpm to separate the supernatant. The whole experiment was conducted in the laboratory of environmental medicine, Shantou University Medical College. For the quantitative analysis of Cd in blood, the peak area was determined by graphite furnace atomic absorption spectrophotometry (Jena ZEEnit 650, Analytik Jena, Jena, Germany), and the injection volume was set to 20 μL. The main parameters of the experiment were as follows: a lamp current of 4.0 mA; a width of 1.2 nm; a wavelength of 228.8 nm; drying temperature at 90, 105, and 120 °C; pyrolyzing and atomization at 300 and 1300 °C, respectively. The limits of detection (LODs) were calculated to be 0.01 μg/L. The results of measurement accuracy showed recoveries within 100 and 103% by analyzing the spiked samples (n = 8). That the precision was better than 5.5%indicated good repeatability for cadmium detection. In order to ensure the quality control and assurance of the experiment, we used standard solutions, analytical duplicates, and reagent blanks to detect drift, precision, and signs of contamination in turn. The linear correlation coefficient of the cadmium standard calibration curve was good, reaching 0.9964. All glassware and plastic in this study were soaked in 20% (v/v) HNO_3_ for over 24 h and rinsed three times with deionized water.

### 2.5. Statistical Analysis

Data were analyzed by using SPSS 23.0 (SPSS Inc., Chicago, IL, USA). Data with normal distribution are represented by the mean ± SD and were analyzed by an independent-sample *t* test. Data with non-normal distribution are represented by median (interquartile range (IQR)) and were analyzed by the Mann–Whitney U test and Kruskal–Wallis H test. Categorical variables were analyzed by the chi-square test. The data for blood Cd level were divided into two groups by median concentration (3.84 mg/L), named high level and low level, respectively. Survival time was defined as the time between the diagnosis of NPC and the discovery of progressive NPC or death. Kaplan–Meier log-rank tests were used to analyze survival time by various factors, and median survival time was also estimated by survival analysis. Progression-free survival (PFS) was analyzed by the variables age at diagnosis; family history of cancer; history of disease; T, M and N classification; clinical stage; EA and VCA positivity; pathological type; and blood Cd level by Cox proportional-hazard regression models, estimating hazard ratios (HRs) and 95% confidence intervals (CIs). Spearman rank correlation analysis was also used to evaluate the relationships between investigated factors and blood Cd level with NPC. All tests were two-sided and *p* < 0.05 was considered statistically significant.

## 3. Results

### 3.1. Association between Clinical Characteristics and NPC Progression

We included 134 patients with NPC (76.9% females; mean age 55.57 ± 11.93 years). The median progression-free survival (PFS) for all patients was 2 months and 49 patients showed NPC progression: eight (16.3%) had a family history of cancers, 35 (71.4%) had a smoking history, and eight (16.3%) had a drinking history (Table 1). The proportion of patients with disease progression was higher with than without a history of smoking (71.4% vs. 28.6%; *p* = 0.018; Table 1). In addition, the proportion of relatively high median blood Cd level was higher with than without disease progression (7.62 (IQR 3.37–9.43) vs. 3.09 (1.77–4.80) μg/L; *p* < 0.001, Table 1).

### 3.2. Association between Clinical Characteristics of NPC Patients and Blood Cd Level

Blood Cd level was related to the history of disease (*p* = 0.017), smoking history (*p* = 0.014), clinical stage (*p* = 0.016), and smoking pack-years (*p* = 0.003) (Table 2). For further analysis, we divided the blood Cd level into high level (≥ 3.84 μg/L) and low level (< 3.84 μg/L) according to the median concentration. Probability of high Cd level was associated with smoking ≥ 40 pack-years versus not smoking (OR = 4.83, 95% CI 1.68–13.91, *p* = 0.004) (Table 3). Probability of low Cd level was associated with history of disease versus no history (OR = 0.39, 95% CI 0.17–0.89, *p* = 0.025). Additionally, probability of high Cd level was associated with advanced disease stage but not significantly.

Blood Cd level was positively correlated with clinical stage (r = 0.192, *p* < 0.05), smoking pack-years (r = 0.314, *p* < 0.01), and smoking history (r = 0.224, *p* < 0.01) (Table 4). We found a negative correlation between blood Cd level and history of disease (r = −0.202; *p* < 0.05).

### 3.3. Prognostic Factors Associated with PFS and NPC Progression

**Figure 1:**
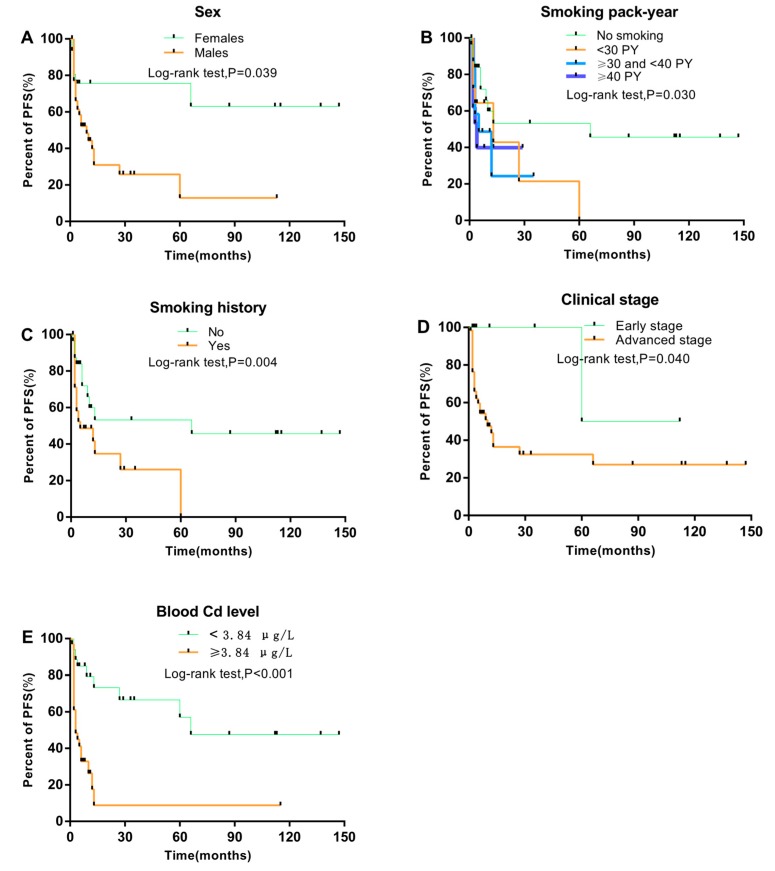
Comparison of PFS among different groups of NPC patients by Kaplan–Meier log-rank test. (**A**–**E**) PFS by sex, smoking pack-years, smoking history, clinical stage, and blood Cd level, respectively (n = 134).

**Figure 2:**
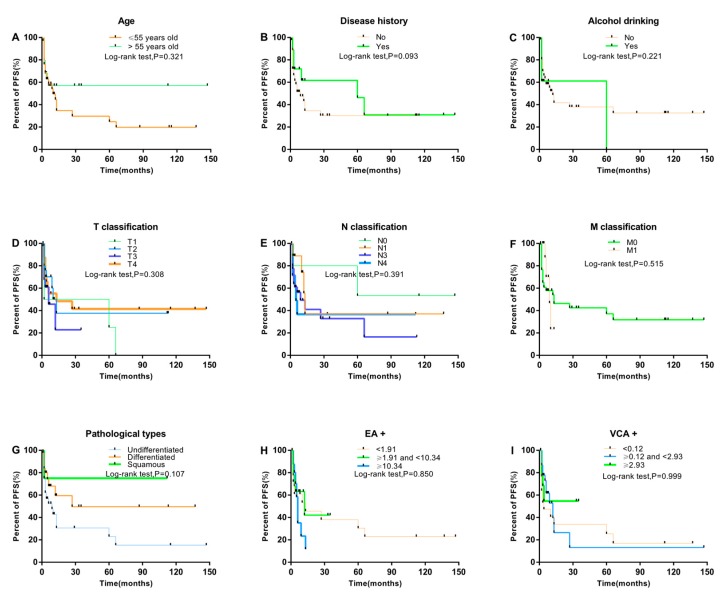
Comparison of PFS among different groups of NPC patients by Kaplan–Meier log-rank test. (**A**–**I**) PFS by age, disease history, alcohol drinking, T classification, N classification, M classification, pathological type, early antigen-positive (EA+), and VCA, viral capsid antigen antibody-positive (VCA+), respectively (n = 134).

Kaplan–Meier analysis showed that smoking history, sex, blood Cd level, smoking pack-years, and clinical stage are all associated with PFS (log-rank test; all *p* < 0.05; Figure 1). PFS was shorter for males than females (*p* = 0.039) and with than without a smoking history (*p* = 0.004). With increasing smoking pack-years and clinical stage, PFS decreased (*p* = 0.031, and 0.040, respectively). Moreover, high blood Cd level was strongly associated with short PFS (*p* < 0.001). Nevertheless, short PFS was not associated with age at diagnosis, family history of cancer, alcohol drinking, pathological type, type of Epstein–Barr virus antibody (EA, VCA), or T, N, M classification (Figure 2).

High blood Cd level was a significant prognostic risk factor for NPC progression (HR = 3.76; 95% CI 1.75–8.06; *p* = 0.001; Table 5).

## 4. Discussion

The present study assessed the prognosis of NPC in patients by analyzing blood Cd level and clinicopathological data. Blood Cd level was positively correlated with N classification, smoking history, and smoking pack-years and negatively with disease history. High blood Cd level, male sex, smoking history and increasing pack-years as well as advanced clinical stage were significantly associated with short PFS. In addition, high blood Cd level was an independent prognostic factor for short PFS. Clinicopathologic factors and blood Cd level may affect the prognosis of NPC, and blood Cd level could be a novel risk factor for NPC progression.

Patient sex, smoking history, and clinical stages were all prognostic factors of NPC, but no association was found with drinking history, pathological type, N classification, and radiotherapy pattern in previous studies [32,39]. Sex can affect the curative effect of NPC patients, and men were found to have a higher rate of metastasis and disease progression than women, which is consistent with our results [40,41]. Sex was an important prognostic factor, and female NPC patients were previously found to have a higher survival rate than males (80.2 and 71.9%) [32]. Smoking is an important factor in tumor development and causes normal epithelial cell tumors to develop as mutagen and DNA damage agents [42]. Several studies have evaluated the effect of tobacco use in head and neck cancer, including NPC. Tobacco use decreased head and neck cancer survival during radiotherapy or medication [43]. A follow-up study showed smoking status as an important predictor of survival in NPC patients [44]. NPC patients who gave up smoking had a higher survival rate than those who currently smoked [45]. Of course, smoking pack-years may also be associated with the survival and prognosis of NPC because smoking time and quantity accumulate.

The 5-year survival rate is higher in the early clinical stages (stages I and II) of NPC patients [46,47]. However, over half of the patients are diagnosed at advanced stages, which greatly reduce the survival rate [48]. The tumor has no obvious symptoms at the early stage; symptoms are mostly observed in the advanced stage. Cheng et al. found that tumor stage is a significant prognostic factor for NPC [33]. Furthermore, clinical stage was previously found related to overall survival of patients [35]. A study of prostate cancer suggested higher Cd levels detected in advanced stages [49]. The advanced stage has a poor prognostic effect on local control rate and metastasis of the disease, whereas early stages have a better survival advantage [33,50].

Many studies have found Cd level as a risk factor for NPC [22,36,37], but few assessed the effect of Cd exposure on the survival and prognosis of NPC. One study investigated a head and neck cancer population in Tunisia, including 48 NPC patients and 97 laryngeal cancer patients; Cd level was higher in NPC patients than controls (2.95 ± 4.93 vs. 0.74 ± 1.15, *p* < 0.001) [22]. Another study, also carried out in Tunisia, found higher Cd level in tumor tissues of patients with head and neck cancer (including 34 NPC patients, 45 laryngeal cancer patients) than in healthy tissues (0.95 ± 1.38 vs. 0.22 ± 0.43, *p* < 0.001) [36]. By adding a normal control population, it will also be significant to explore blood Cd level on the risk of NPC. Actually, we conducted a case-control study [37] previously to explore the association between Cd level and NPC risk, and found that Cadmium seemed to be a risk factor of NPC, and that high Cd exposure may promote the occurrence and development of NPC.These findings were relatively consistent. In the present study, we assessed the association between these factors and PFS with NPC for a better understanding of the prognostic process. Cd, as a novel risk factor, should be specifically analyzed for its effect on NPC prognosis.

With the industrial production process, Cd (a common toxic metal pollutant) exists in water, soil, food and air [24]. Cd was first identified as a lung carcinogen in mechanism and epidemiological studies, and it exists widely in soft tissue [51]. Cd has certain toxic effects on breast, prostate, kidney and bone tissue by external ingestion and body accumulation [52]. Recent studies have also found high levels of blood Cd in patients with nasosinusal polyposis and NPC. In addition, in a case-control study, blood Cd level was higher in NPC cases than controls (OR = 3.42; 95% CI 1.86–6.30) [37]. Another study concluded high blood Cd level in cases [22], and the result was consistent with our previous research [37]. In this study, we found that high Cd level was related to N classification, clinical stage, smoking history and disease history in patients with NPC. High blood Cd level was significantly related to NPC progression, and Cd exposure may be associated with NPC prognosis.

Here, we found that high Cd level was related to short PFS. Moreover, multivariate analysis concluded Cd as an independent prognostic factor for NPC progression after adjustment for other factors, including sex, residence, family history of cancer, history of disease, alcohol drinking, smoking pack-years, clinical stage, and blood Cd level. Previous studies have found that Cd level isa risk factor for NPC development and is also associated with the prognosis of NPC [22].

There are many sources of metal exposure, and ingestion and inhalation are two major ways for metals to enter the body. Cd toxicity is well known and has a long half-life of approximately 20 years in an organism, so the concentration in body fluids must be monitored [53]. Kidney is the main accumulation organ of Cd, but other soft tissues also show a certain accumulation effect, such as the prostate, lungs, and mucosa. Hence, the elevated incidence of respiratory and renal disease with Cd exposure is not surprising [54]. Cd has been classified as a carcinogen to humans by the International Agency for Research on Cancer for a long time, and relevant experiments have shown that subcutaneous injection of Cd into rodents causes local sarcomata formation [55,56,57]. One study found that battery workers had a high risk of NPC, caused by the long-term inhalation of large amounts of Cd particles that accumulate in the nose [54]. Anosmia is a kind of toxic disease caused by long-term exposure to high Cd levels [58]. Recent evidence showed that Cd entering the body mainly interfered with the gene synthesis and repair process, thus affecting the response to adverse effects and might contribute to chronic nasal inflammation [59]. Exposure to Cd in the external environment exceeds immunology function and results in tumor formation, and the process may explain the progression of NPC [60].

Because of Cd exposure and accumulation in the body, the respiratory function and metabolism of the body are changed. With exposure to different chemical poisons, the involved genes have different detoxification mechanisms, such as the enzyme cytochrome P450 (CYP2E1). The activity of CYP2E1 increased in mucosal cells induces the formation of free radicals and leads to cell injury. Additionally, it may interfere with gene regulation and cell differentiation. Because of different repair mechanisms of DNA, abnormal gene expression that encodes cell growth promotes the occurrence of tumors [22,61]. Normally, NPC occurs from the combined action of various mixtures, such as other metals or organic chemicals. Other studies have found that elevated blood levels of Cd result from an imbalance in Zn/magnesium ratio [62]. Several studies suggested a higher blood Cd level in NPC patients than healthy controls (*p* < 0.05) [22,37], so Cd could be a risk factor for NPC patients and may also affect the prognosis of patients and increase the chances of NPC progression.

Apart from occupational and environmental exposures, lifestyle habits such as smoking are a major factor in Cd intake. Many studies have shown that tobacco smoke exposure increases Cd level in the body [63,64], and the Cd level is approximately 2 to 4 times higher in smokers than non-smokers [65]. We also found a strong positive correlation between Cd level and smoking history. Similarly, blood Cd level was significantly correlated with the number of daily cigarettes smoked and pack-years of smoking. Cd exposure is much higher from cigarette smoking than diet and air. In addition, we found that smoking pack-years isa risk factor of blood Cd level (OR = 4.83; 95% CI 1.68–13.91; Table 3). Of note, the median blood Cd level was previously found much higher in smokers than non-smokers (1.02 vs. 0.51 ng/mL) [63]. In our study, the blood Cd level was higher in smokers than non-smokers (*p* = 0.01). Smoking history was associated with short PFS on Kaplan–Meier but not multivariate analysis. Blood Cd level may mask the true effects of smoking. Further studies are needed to clarify whether smoking affects the prognosis of NPC.

We found that Cd, as a novel risk factor, affected the prognosis of NPC, which indicates the danger of behaviors and environmental metal exposure to the human body to some extent. Intervening in Cd exposure may better guide patients to better prognosis and clinical practice. However, our study contains some limitations, such as the confounding effect of clinical stage on prognosis, and other factors that may have contributed to biased results. Firstly, this was not a case-control study, but a survival analysis of factors affecting the prognosis of NPC cases. We included NPC cases and observed the occurrence of endpoint events during follow-up, so the proportion of outcomes and non-outcomes could not be designed in advance. Due to the limited sample size, the results may be slightly biased. Further intensive studies based on well-designed methods and larger sample size are needed to examine the association. Secondly, although we did not collect accurate information about the dose of chemotherapy using cisplatin and paclitaxel on NPC patients, the effect of chemotherapy on the findings was less likely. Since all the patients we included received similar chemotherapy, this is most likely to have resulted in the attenuation of risk estimates toward the null between two groups. Hence, the results of the study were unaffected and reliable. Certainly, the effect of platinum (Pt) used for chemotherapies on the prognosis of NPC patients also needsto be explored in future studies. Thirdly, to better reflect the effects of long-term and chronic exposure to metals, the sample tested should be urine; blood Cd is rapidly cleared by the kidneys, so it is often used as a biomarker of recent exposure [66]. However, high blood Cd level and long-term exposure are relatively consistent, so the level can also be used to reflect past exposure [67,68]. Therefore, the use of blood Cd level can reflect the human burden of Cd.

## 5. Conclusions

In the present study, we assessed the effects of various factors on the prognosis and progression of NPC by measuring blood Cd level and clinicopathologic information. Cd level was an independent prognostic risk factor for NPC on multifactorial Cox regression analysis. Furthermore, high blood Cd level, male sex, smoking history and increasing pack-years as well as clinical stage were risk factors for short PFS with NPC. Subsequent studies are needed to clarify the effect of Cd exposure and other clinical factors on the prognosis and the progression of NPC.

## Figures and Tables

**Table 1:** Clinicopathologic characteristics of the participants with and without nasopharyngeal carcinoma (NPC) progression (n = 134).

Clinicopathologic Characteristics	NPC Progression n = 49	No NPC Progression n = 85	*p*-Value
Age (years), mean ± SD	54.00 ± 11.61	56.47 ± 12.08	0.250 ^a^
Cd level (μg/L), median, IQR	7.62 (3.37–9.43)	3.09 (1.77–4.80)	<0.001 *
EA+	4.53 (0.73–10.08)	5.99 (2.51–10.36)	0.270 ^a^
VCA+	0.37 (0.03–2.05)	1.15 (0.21–3.09)	0.092 ^a^
Sex			
Female	8 (16.3)	23 (27.1)	0.156 ^b^
Male	41 (83.7)	62 (72.9)	
Family history of cancer			
Yes	8 (16.3)	15 (17.6)	0.845 ^b^
No	41 (83.7)	70 (82.4)	
History of disease			
Yes	12 (24.5)	33 (38.8)	0.091 ^b^
No	37 (75.5)	52 (61.2)	
Smoking pack-years			
No smoking	14 (28.6)	42 (49.4)	0.118 ^b^
<30	12 (24.5)	17 (20.0)	
30–40	8 (16.3)	10 (11.8)	
≥40	15 (30.6)	16 (18.8)	
Smoking history			
Yes	35 (71.4)	43 (50.6)	0.018 ^b^
No	14 (28.6)	42 (49.4)	
Alcohol drinking			
Yes	8 (16.3)	10 (11.8)	0.456 ^b^
No	41 (83.7)	75 (88.2)	
T classification			
T1	5 (10.4)	1 (1.2)	0.080 ^b^
T2	14 (29.2)	32 (37.6)	
T3	13 (27.1)	20 (23.5)	
T4	16 (33.3)	32 (37.6)	
Clinical stages			
Early	1 (2.0)	8 (9.4)	0.101 ^b^
Advanced	48 (98.0)	77 (90.6)	
N classification			
N0	2 (4.2)	3 (3.5)	0.573 ^b^
N1	5 (10.4)	14 (16.5)	
N2	34 (70.8)	61 (71.8)	
N3	7 (14.6)	7 (8.2)	
M classification			
M0	44 (91.7)	77 (90.6)	0.835 ^b^
M1	4 (8.3)	8 (9.4)	
Pathological types			
Undifferentiated	36 (73.5)	50 (58.8)	0.234 ^b^
Differentiated	12 (24.5)	32 (37.6)	
Squamous	1 (2.0)	3 (3.5)	

Smoking pack-years, [cigarettes per day/20] × years smoked; T classification, size of the primary tumor; N classification, lesion of regional lymph nodes; Cd, cadmium; IQR, interquartile range; EA, early antigen; VCA, viral capsid antigen antibody. * Mann–Whitney U test due to non-normal distribution; ^a^ Independent sample *t* tests; ^b^ chi-square test.

**Table 2:** Association between clinicopathological features and blood Cd levels with NPC (n = 134).

Clinicopathologic Characteristics	No. of Patients	Blood Cd Level		*p*-Value
		<3.84 μg/L (n = 67)	≥3.84 μg/L (n = 67)	
Age (years), mean ± SD	134	55.40 ± 11.66	55.73 ± 12.28	0.631 ^a^
EA+	106	5.13 (1.73–9.97)	7.10 (2.49–10.71)	0.185 ^a^
VCA+	107	0.81 (0.12–2.40)	0.89 (0.21–3.35)	0.432 ^a^
Sex				
Female	103	49 (73.1)	54 (80.6)	0.306^b^
Male	31	18 (26.9)	13 (19.4)	
Family history of cancer				
Yes	23	8 (11.9)	15 (22.4)	0.109 ^b^
No	111	59 (88.1)	52 (77.6)	
Disease history				
Yes	45	29 (43.3)	16 (23.9)	0.017 ^b^
No	89	38 (56.7)	51 (76.1)	
Smoking pack-years				
No smoking	56	35 (52.2)	21 (31.3)	0.003 ^b^
＜30	29	17 (25.4)	12 (17.9)	
30–40	18	8 (11.9)	10 (14.9)	
≥40	31	7 (10.4)	24 (35.8)	
Smoking history				
Yes	78	32 (47.8)	46 (68.7)	0.014 ^b^
No	56	35 (52.2)	21 (31.3)	
Alcohol drinking				
Yes	18	7 (10.4)	11 (16.4)	0.311 ^b^
No	116	60 (89.6)	56 (83.6)	
Clinical stage				
Early	9	8 (11.9)	1 (1.5)	0.016 ^b^
Advanced	125	59 (88.1)	66 (98.5)	
T classification				
T1	6	2 (3.0)	4 (6.1)	0.356 ^b^
T2	46	25 (37.3)	21 (31.8)	
T3	33	13 (19.4)	20 (30.3)	
T4	48	27 (40.3)	21 (31.8)	
N classification				
N0	5	3 (4.5)	2 (3.0)	0.345 ^b^
N1	19	13 (19.4)	6 (9.1)	
N2	95	45 (67.2)	50 (75.8)	
N3	14	6 (9.0)	8 (12.1)	
M classification				
M0	121	62 (92.5)	59 (89.4)	0.527 ^b^
M1	12	5 (7.5)	7 (10.6)	
Pathological types				
Undifferentiated	86	40 (59.7)	46 (68.7)	0.410 ^b^
Differentiated	44	24 (35.8)	20 (29.9)	
Squamous	4	3 (4.5)	1 (1.5)	

Smoking pack-years, [cigarettes per day/20] × years smoked; T classification, size of the primary tumor; N classification, lesion of regional lymph nodes; Cd, cadmium; EA, early antigen; VCA, viral capsid antigen antibody. ^a^ Independent sample *t* tests; ^b^ chi-square test.

**Table 3:** Multivariate analysis of factors associated with blood Cd level for NPC patients (n = 134).

Variables	Blood Cd Level		OR (95％CI)	*p*-Value
	<3.84 μg/L n = 67	≥3.84 μg/L n = 67		
Family History of Cancer				
No	59 (88.1)	52 (77.6)	Reference	
Yes	8 (11.9)	15 (22.4)	1.52 (0.53–4.40)	0.437
Smoking pack-years				
No smoking	35 (52.2)	21 (28.8)	Reference	
<30	17 (25.4)	12 (16.4)	1.14 (0.44–2.96)	0.790
30–40	8 (11.9)	16 (21.9)	2.01 (0.64–6.32)	0.228
≥40	7 (10.4)	24 (32.9)	4.83 (1.68–13.91)	0.004
History of disease				
No	38 (56.7)	51 (76.1)	Reference	
Yes	29 (43.3)	16 (23.9)	0.39 (0.17–0.89)	0.025
Clinical stage				
Early	8 (11.9)	1 (1.5)	Reference	
Advanced	59 (88.1)	66 (98.5)	5.50 (0.62–48.54)	0.125

OR, odds ratio; 95% CI, 95% confidence interval; Smoking pack-years, [cigarettes per day/20]×years smoked.

**Table 4:** Spearman correlation coefficients between investigated factors and blood Cd level with NPC (n = 134).

Investigated Factors	SPY	Age	Sex	FHC	DH	AD	CS	PT	SH	BCL	VCA+
EA+	−0.025	0.204 *	−0.108	−0.045	−0.129	−0.268 **	0.032	0.207 *	−0.080	0.167	0.476 **
VCA+	-0.082	0.149	0.179	−0.039	−0.014	−0.066	−0.095	−0.105	−0.100	0.065	
BCL	0.314 **	−0.006	−0.056	0.146	−0.202 *	−0.088	0.192 *	−0.155	0.224 **		
SH	0.899 **	0.064	−0.612 **	0.145	−0.006	0.289 **	0.135	0.017			
PT	0.031	0.017	−0.074	0.020	−0.051	−0.100	−0.003				
CS	0.143	−0.064	−0.065	0.122	−0.125	0.018					
AD	0.269 **	0.048	−0.216 *	0.111	0.183*						
DH	−0.023	0.056	−0.053	0.137							
FHC	0.241 **	0.107	−0.109								
Sex	−0.539 **	−0.039									
Age	0.204 *										

SPY, smoking pack-year; FHC, family history of cancer;DH, history of disease; AD, alcohol drinking; CS, clinical stage; PT, pathological type; SH, smoking history; BCL, blood Cd level; EA, early antigen; VCA, viral capsid antigen antibody.*p < 0.05; **p < 0.01.

**Table 5:** Proportional-hazards analysis of factors associated with progression-free survival (PFS) with NPC (n = 134).

Variables	PFS		
	HR	95% CI	*p*-Value
Sex (male vs. female)	1.25	0.45–3.42	0.671
Residence			
Chaozhou	Reference		
Shantou	0.70	0.28–1.74	0.443
Jieyang	0.99	0.39–2.48	0.976
Other area	0	0	0.976
Family history of cancer (yes vs. no)	1.11	0.49–2.53	0.800
History of disease (yes vs. no)	0.65	0.31–1.34	0.240
Alcohol drinking (yes vs. no)	1.11	0.46–2.71	0.817
Smoking pack-years			
No smoking	Reference		
<30	2.32	0.87–6.22	0.093
30–40	1.47	0.52–4.18	0.468
≥40	1.76	0.70–4.42	0.230
Clinical stage (advanced vs early)	3.03	0.37–24.65	0.301
Blood Cd level (≥3.84 vs. <3.84 μg/L)	4.11	1.92–8.81	<0.001

HR, hazard ratio; Smoking pack-years, [cigarettes per day/20] × years smoked.
